# Transmembrane proteoglycans syndecan-2, 4, receptor candidates for the impact of HGF and FGF2 on semaphorin 3A expression in early-differentiated myoblasts

**DOI:** 10.14814/phy2.12553

**Published:** 2015-09-17

**Authors:** Mai-Khoi Q Do, Naomi Shimizu, Takahiro Suzuki, Hideaki Ohtsubo, Wataru Mizunoya, Mako Nakamura, Shoko Sawano, Mitsuhiro Furuse, Yoshihide Ikeuchi, Judy E Anderson, Ryuichi Tatsumi

**Affiliations:** 1 Department of Animal and Marine Bioresource Sciences, Kyushu UniversityFukuoka, Japan; 2 Graduate School of Agriculture, Kyushu UniversityFukuoka, Japan; 3 Department of Biological Sciences, Faculty of Science, University of ManitobaWinnipeg, Manitoba, Canada

**Keywords:** Basic fibroblast growth factor, hepatocyte growth factor, proteoglycans, semaphorin 3A, syndecans

## Abstract

Regenerative mechanisms that regulate intramuscular motor innervation are thought to reside in the spatiotemporal expression of axon-guidance molecules. Our previous studies proposed an unexplored role of resident myogenic stem cell (satellite cell)-derived myoblasts as a key presenter of a secreted neural chemorepellent semaphorin 3A (Sema3A); hepatocyte growth factor (HGF) and basic fibroblast growth factor (FGF2) triggered its expression exclusively at the early differentiation phase. In order to advance this concept, the present study described that transmembrane heparan/chondroitin sulfate proteoglycans syndecan-2, 4 may be the plausible receptor candidates for HGF and FGF2 to signal Sema3A expression. Results showed that mRNA expression of syndecan-2, 4 was abundant (two magnitudes higher than syndecan-1, 3) in early-differentiated myoblasts and their in vitro knockdown diminished the HGF/FGF2-induced expression of Sema3A down to a baseline level. Pretreatment with heparitinase and chondroitinase ABC decreased the HGF and FGF2 responses, respectively, in non–knockdown cultures, supporting a possible model that HGF and FGF2 may bind to heparan and chondroitin sulfate chains of syndecan-2, 4 to signal Sema3A expression. The findings, therefore, extend our understanding that HGF/FGF2-syndecan-2, 4 association may stimulate a burst of Sema3A secretion by myoblasts recruited to the site of muscle injury; this would ensure a coordinated delay in the attachment of motoneuron terminals onto fibers early in muscle regeneration, and thus synchronize the recovery of muscle fiber integrity and the early resolution of inflammation after injury with reinnervation toward functional recovery.

## Introduction

Myogenesis and remodeling of neuromuscular connections are both critical for restoring muscle contractile properties during muscle regeneration. While the coordination of these two processes remains unclear, various neural factors including attractive and repulsive axon-guidance cue ligands and their receptors may be involved (McLoon 2009; Ten Broek et al. 2010; Turner and Badylak 2012).

Recently, we found that satellite cells, resident myogenic stem cells found beneath the basal lamina in high density at neuromuscular junctions of mature fibers (Kelly 1978; Wokke et al. 1989; Bischoff and Franzini-Armstrong 2004), upregulate a secreted potent neural chemorepellent, semaphorin 3A (Sema3A, also referred to as SemaIII, SemD, and collapsing) (Tanelian et al. 1997; Goodman et al. 1999; Kuhn et al. 1999; Kawasaki et al. 2002; Ieda et al. 2007; Moret et al. 2007) in the early differentiation phase of gastrocnemius muscle regeneration after injury by crush or cardiotoxin injection (Tatsumi et al. 2009; Sato et al. 2013). Sema3A was upregulated 4 days postinjury and plateaued at a high level (about 16-fold more than baseline) 6–12 days postinjury before returning to baseline. Importantly, we showed upregulation of Sema3A expression and secretion by hepatocyte growth factor (HGF) and basic fibroblast growth factor (FGF2) in primary satellite cell (myoblast) cultures early in differentiation and in satellite cells on fibers (Tatsumi et al. 2009; Do et al. 2011; Suzuki et al. 2013). Both effects were prevented by adding transforming growth factor (TGF)-*β*2, 3 in culture (Do et al. 2011). Similarly, in cultures of the mouse C2C12 myoblasts, Sema3A secretion was upregulated 2 days after switching to differentiation media and returned to baseline 3 days later (Henningsen et al. 2010). The physiological significance of these findings was confirmed by in vivo experiments of growth factors in extracellular wound fluid that found active HGF and FGF2 prevalent 4–6 days after crush injury of rat gastrocnemius muscle, while TGF-*β*3 increased at 12 days (Do et al. 2012). These results emphasized a plausible role for HGF and FGF2 in the time-coordinated upregulation of Sema3A expression that could ensure the chemorepulsive events necessary to synchronize a delay in neurite sprouting and attachment of motoneuron terminals onto regenerating fibers. However, there is still a critical need to explore the molecular mechanisms of HGF/FGF2-induced Sema3A upregulation.

**Figure 1:**
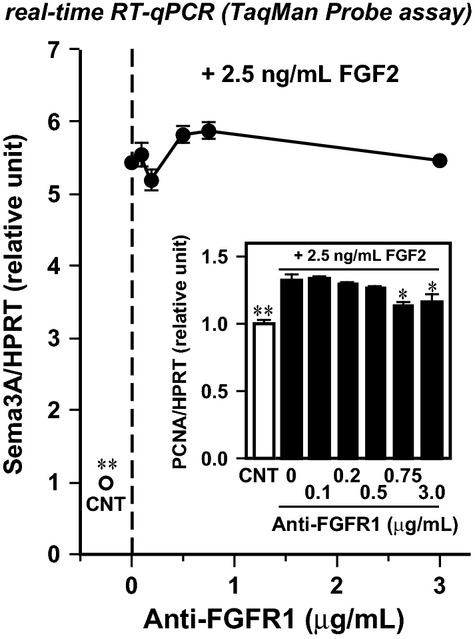
Null effect of FGFR1 immunoneutralization on FGF2-induced Sema3A upregulation. Satellite cells (over 95% desmin-positive at 30 h postplating) were isolated from back and upper hind-limb muscles of 9-month-old male Sprague Dawley rats according to Allen et al. (1997) and maintained in DMEM-10% HS for 48 h. Myoblasts were then pretreated with 0–3.0 *μ*g/mL mouse monoclonal anti-FGFR1 neutralizing antibody (LS-C23657; Lifespan BioSciences, Washington, DC) (Kang et al. 2012) in DMEM-10% HS for 90 min prior to adding recombinant FGF2 (2.5 ng/mL) to the media. Cells were maintained for the next 24 h, and evaluated for Sema3A expression (closed circle) by real-time RT-qPCR run under the TaqMan probe detection format, standardized to the expression of HPRT. Inset, PCNA expression in the same cultures; note that the cell proliferation activity was reduced by the immunoneutralization even in the presence of FGF2 (solid bar), and hence this treatment served as an important positive control. Open circle and bar labeled “CNT” represent the negative control culture without the neutralizing antibody or FGF2 addition. Data points and bars depict the means ± SEs for three cultures per treatment and significant differences from the positive control culture that received 2.5 ng/mL FGF2 (without anti-FGFR1 antibody) at *P *< 0.05 and *P *< 0.01 are indicated by (*) and (**), respectively.

**Figure 2:**
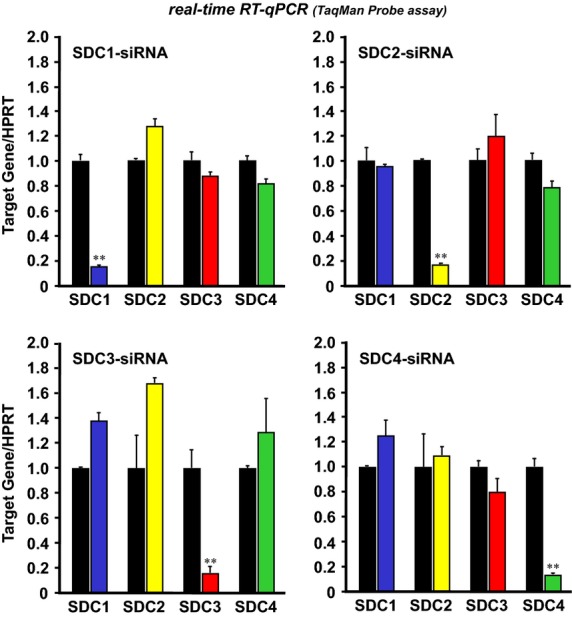
Knockdown potential of SDC isoform-specific siRNAs in myoblast cultures. Myoblast cultures were transfected with each siRNA at 100 nmol/L, as follows: control siRNA (black bars) and SDC1–4 siRNA (colored bars; Stealth_384, 715, 789, and 251, respectively) in DMEM-5% HS without HGF/FGF2, followed by evaluation of the level of expression of SDC1–4 mRNA at 24 h by real-time qPCR standardized to HPRT. Data bars depict the means ± SEs for three cultures per treatment; significant differences from control siRNA culture means at *P* < 0.01 are indicated by (**).

The present study was designed to examine membrane receptor(s) for HGF and FGF2 that signal the burst in Sema3A expression at the early differentiation phase of myoblasts. Since HGF/FGF2 responses share dose and timing dependencies (see Figs. 2A, B and 3B in Tatsumi et al. 2009 and Figs. 1A, B and 2 in Do et al. 2011), both growth factors may share a common binding/signaling receptor(s), although a receptor using protein–protein interactions for ligand binding is unlikely since HGF and FGF2 proteins do not have significant sequence homology. This idea is supported by our previous observation that tyrosine kinase c-met, a specific receptor for HGF, was excluded as mediating this signaling pathway, since treatment with anti-c-met neutralizing antibody did not affect HGF-induced Sema3A upregulation (see Fig. 5 in Tatsumi et al. 2009). As well, our pilot study showed that neutralizing antibody for FGF receptor-1 (FGFR1, most abundant in activated satellite cells; Kästner et al. 2000; Sheehan and Allen 1999) also did not diminish Sema3A upregulation, suggesting FGFRs may not mediate FGF2 signaling (see Fig. 1). In addition to these canonical high-affinity receptors, HGF and FGF2 are also known to bind to glycosaminoglycan (GAG) chains of proteoglycans (Carey 1997; Rapraeger 2000; Deepa et al. 2004; Asada et al. 2009; Xian et al. 2010) that mediate important functions of the extracellular matrix. Our primary hypothesis here was that the association between HGF/FGF2 and heparan/chondroitin sulfate chains of the transmembrane proteoglycan, syndecan (SDC), impacts the Sema3A expression.

SDCs are members of a multigene family with overlapping, but nonequivalent functions. SDC proteins have an *N*-terminal ectodomain of a core protein modified by some distinct chondroitin sulfate and/or heparan sulfate chains, a conserved single-pass hydrophobic transmembrane domain, and a short *C*-terminal cytoplasmic domain containing four well-conserved tyrosine residues as possible phosphorylation sites (Carey 1997; Multhaupt et al. 2009; Xian et al. 2010; Choi et al. 2011). SDCs are grouped into two subfamilies of SDC1, 3 and SDC2, 4 based on the sequence homology of core proteins and now considered to have important roles in development, wound healing, inflammation, and tumor progression (Carey 1997; Oh and Couchman 2004; Xian et al. 2010; Choi et al. 2011; Elfenbein and Simons 2013) and in satellite cell biology and signaling during muscle development and regeneration (Cornelison et al. 2001, 2004; Casar et al. 2004; Pisconti et al. 2010; Do et al. 2011; Brandan and Gutierrez 2013).

The present study showed that SDC2 and 4 are highly abundant in early-differentiated myoblasts (two magnitudes higher than SDC1 and 3) and in vitro knockdown treatments of SDC2 and 4 abolished HGF/FGF2-induced upregulation of Sema3A. Findings suggest a promising role for SDC2 and 4 as membrane receptors of HGF and FGF2 that may mediate Sema3A expression signaling in early-differentiated myoblasts.

## Materials and Methods

### Animal care and use

All experiments involving the animals were conducted in strict accordance with the recommendations in the Guidelines for Proper Conduct of Animal Experiments published by the Science Council of Japan and with the ethical approval of Kyushu University Institutional Review Board (approval no. A21-117-0 and A22-148-0). Animals were housed at 22 ± 2°C and 55 ± 10% humidity on a 12:12 h light/dark cycle (lights on at 8 am), and received de-ionized water and CRF-1 diet ad libitum during an over 1-week equilibration period.

### Knockdown cultures

A satellite cell-derived myoblast preparation (100% desmin-positive at 48 h postplating), established from adult C57BL/6J mouse muscles and well characterized for normal myogenic properties by Ojima et al. (2004, 2007, 2014, 2015), was a generous gift from Dr. Koichi Ojima, NARO Institute of Livestock and Grassland Science (Tsukuba, Ibaraki, Japan); it was used in cultures transfected with small interfering RNA (siRNA). Briefly, myoblasts were maintained for 48 h in Ham’s F10 Nutrient Mixture medium (11550-043; Invitrogen, Grand Island, NY) containing 20% FBS (10437-028 from Invitrogen) without addition of any antibiotic or antimycotic, on six-well plates (140675; Thermo Fisher Scientific, Waltham, MA) coated with collagen type I (Cellmatrix Type I-P; Nitta Gelatin, Osaka, Japan), in a humidified atmosphere of 5% CO_2_ at 37°C. After 48 h, cultures were transfected with 100 nmol/L siRNA for the next 72 h using DharmaFECT siRNA Transfection Reagent 1 (T-2001-01; Thermo Fisher Scientific) in a differentiation medium of 5% normal horse serum (HS; 16050-122 from Invitrogen) in high-glucose type DMEM (11995-065 from Invitrogen; pH 7.2), according to the manufacturer’s recommendation. One hour postsiRNA transfection (see a scheme shown in Fig. 4A for culture design), differentiating cultures also received 25 ng/mL recombinant mouse HGF (2207-HG; R&D Systems, Minneapolis, MN) or 2.5 ng/mL rat FGF2 (3339-FB; R&D Systems). Doses of HGF and FGF2 were optimized for their maximum activity to stimulate Sema3A expression in satellite cell cultures, as described previously (Tatsumi et al. 2009; Do et al. 2011). Cultures that underwent transfection with Allstars Negative Control siRNA (1027281; Qiagen, Hilden, Germany) served as the control group. SDC siRNAs used here were Stealth RNAi^–^ duplexed oligos (Invitrogen) that were designed with BLOCK-iT^–^ RNAi Designer and formulated specifically to block the target mouse SDC transcripts. The sense and antisense strands of the three nonoverlapping Stealth siRNAs against SDC1–4 are described in Table 1. Stealths_384, 715, 789, and 251 against SDCs1, 2, 3, and 4, respectively, were chosen for the present study; they demonstrated over 80% silencing potential for mRNA expression at 100 nmol/L, assayed at 24 h postdifferentiation in the absence of HGF and FGF2 (Fig. 2). Other sequences did not efficiently suppress the target RNA expression or showed toxicity, and therefore were not used for experiments. Importantly, siRNAs for SDC2, 4 did not significantly decrease the levels of SDC1, 3 expression and vice versa, at 24 h transfection (Fig. 2). This observation confirmed that the effects of specific knockdown in individual SDC1–4 siRNA cultures were not confounded by substitutive alteration in expression of one or more nontargeted SDCs.

**Table 1:** Stealth siRNA strands for mouse SDC1–4

siRNA against	Stealth RNAi*	Sequence (5′ to 3′)
SDC1	Stealth_384†	Sense CACAGGUGCUUUGCCAGAUACUUUG
		Antisense CAAAGUAUCUGGCAAAGCACCUGUG
	Stealth_813	Sense AUUGGCAGUUCCAUCCUCGACAACC
		Antisense GGUUGUCGAGGAUGGAACUGCCAAU
	Stealth_851	Sense GAGAGGGCUCUGGAGAACAAGACUU
		Antisense AAGUCUUGUUCUCCAGAGCCCUCUC
SDC2	Stealth_715†	Sense UUGGGAUGUUGUCAGAACUGGACUC
		Antisense GAGUCCAGUUCUGACAACAUCCCAA
	Stealth_790	Sense AAUGCUUUGUGUCUUCAACGUCAUG
		Antisense CAUGACGUUGAAGACACAAAGCAUU
	Stealth_952	Sense GACAGAAGUUCUAGCAGCCGUCAUU
		Antisense AAUGACGGCUGCUAGAACUUCUGUC
SDC3	Stealth_578	Sense GGUCGUAGAAGAGUCCAGCCAGAAA
		Antisense UUUCUGGCUGGACUCUUCUACGACC
	Stealth_789†	Sense ACAGCUGCCACAGCCAAGAUCACUA
		Antisense UAGUGAUCUUGGCUGUGGCAGCUGU
	Stealth_936	Sense GAGCCUGAUGUUGCUGAGAGGAGUA
		Antisense UACUCCUCUCAGCAACAUCAGGCUC
SDC4	Stealth_92	Sense ACCUCUGUCUCUCGAAUCGACUCUC
		Antisense GAGAGUCGAUUCGAGAGACAGAGGU
	Stealth_251†	Sense UCCCUGAAGUGAUUGAGCCCUUGGU
		Antisense ACCAAGGGCUCAAUCACUUCAGGGA
	Stealth_452	Sense GAACUGAGGUCUUGGCAGCUCUGAU
		Antisense AUCAGAGCUGCCAAGACCUCAGUUC

* Stealth RNAi^–^ duplexed oligos designed with BLOCK-iT^–^ RNAi Designer (Invitrogen).

† Selected for syndecan isoform knockdown experiments.

### Real-time RT-qPCR

Total RNA was isolated from cultured myoblasts using an RNeasy Micro kit (74004; Qiagen) according to the manufacturer’s recommendation. Tissues of 10-week-old male C57BL/6J mice (purchased from Nihon CLEA, Tokyo, Japan) were subjected to a standard Trizol–chloroform protocol for RNA isolation. cDNA was synthesized from 0.6 to 1.8 *μ*g of total RNA by a reverse-transcriptase SuperScript III (18080-044; Invitrogen) using oligo(dT) primer (H09876; Roche, Mannheim, Germany). The levels of mRNA expression of mouse SDC1, 2, 3, and 4 (accession no. NM_011519.2, 008304.2, 011520.3, and 011521.2, respectively) and Sema3A (NM_009152.3) were monitored by real-time quantitative PCR (qPCR) using a Roche LightCycler1.5 run under the TaqMan probe detection format (Roche) standardized against the expression of hypoxanthine guanine phosphoribosyl transferase (HPRT; accession no. NM_013556.2) or *β*-actin (NM_007393.3). All primer sets were designed using a Roche Universal ProbeLibrary Assay Design Center (ProbeFinder version 2.35) with an intron-spanning assay, as described in Table 2 (the corresponding TaqMan probe numbers included). Annealing temperature was set to 60°C in all cases; the PCR conditions were optimized so that the amplification reactions are within the linear range. If necessary, qPCR data were analyzed by the 2^–ΔΔC(t)^ method (Livak and Schmittgen 2001; Pfaffl 2001) to compare the relative mRNA expression among different target genes (SDC1–4), referenced to *β*-actin expression. E-values were over 0.8 in all cases, indicating that the amplification efficiencies of both target and internal standard genes were good enough for the analysis.

**Table 2:** qPCR primer sets for Sema3A, SDC1–4, and references

Gene	Sequence (5′ to 3′)	Amplicon (nt)	Probe No.[Table-fn tf2-1]
(A) For mouse genes (Figs. 5)			
Sema3A	Forward ATCAGTGGGTGCCTTACCAA	71	72
	Reverse GCCAAATGTTTTACTGGGACA		
SDC1	Forward TCTGGGGATGACTCTGACAAC	68	21
	Reverse TGCCGTGACAAAGTATCTGG		
SDC2	Forward TTCAGGAGTATATCCTATTGATGATGA	76	29
	Reverse ACTCTCTATGTCTTCATCAGCTCCT		
SDC3	Forward GAGGAGTACCCTGCCGTTG	71	92
	Reverse ACTCTGGAGTTGGGGTCTGA		
SDC4	Forward CCCTTCCCTGAAGTGATTGA	98	64
	Reverse AGTTCCTTGGGCTCTGAGG		
HPRT	Forward TCCTCCTCAGACCGCTTTT	90	95
	Reverse CCTGGTTCATCATCGCTAATC		
β-actin	Forward CTAAGGCCAACCGTGAAAAG	104	64
	Reverse ACCAGAGGCATACAGGGACA		
(B) For rat genes (Figs. 1 and 6)			
Sema3A	Forward TTTCAGCAATGGAGCTTTCTACT	82	67
	Reverse CGGTGTAGAGGAAGCTGTGC		
PCNA	Forward TGAACTTTTTCACAAAAGCCACT	103	94
	Reverse TGTCCCATGTCAGCAATTTTA		
HPRT	Forward GACCGGTTCTGTCATGTCG	61	95
	Reverse ACCTGGTTCATCATCACTAATCAC		

TaqMan probes purchased from Roche.

### ECL-western blotting

Whole cell lysates from myoblast cultures were applied to SDS–10% PAGE under reducing conditions and proteins were transferred onto Amersham Hybond ECL nitrocellulose membranes (RPN2020D; GE Healthcare, Little Chalfont, UK) as described previously (Tatsumi et al. 2009). The blots were blocked with 10% powdered milk in 0.1% Tween 20–Tris buffered saline (TTBS) before overnight incubation at 4°C with affinity-purified rabbit polyclonal anti-Sema3A antibody (Chemicon brand AB9604; Merck Millipore, Temecula, CA; 1:500 dilution) and mouse monoclonal anti-*β*-actin antibody (clone AC-15; ab6276 from Abcam, Cambridge, UK; 1:2000 dilution) in CanGetSignal solution 1 (NKB-101; Toyobo, Osaka, Japan). Membranes were subsequently incubated for 1 h with biotinylated anti-rabbit IgG secondary antibody (BA-1000; Vector Lab., Burlingame, CA) at 1:5000 dilution in CanGetSignal solution 2, then with horseradish peroxidase (HRPO)-labeled avidin (PK-6100; Vector Laboratories) at 1:500 dilution in TTBS for 30 min at room temperature. For *β*-actin detection, membranes were incubated in HRPO-conjugated anti-mouse IgG (P0447; Dako, Glostrup, Denmark) at 1:5000 dilution in CanGetSignal solution 2. Finally, immunoreactive bands were visualized using an Amersham enhanced chemiluminescence (ECL) detection kit (RPN2106; GE Healthcare) to expose Amersham Hyperfilm ECL (28906837; GE Healthcare).

### Statistical analysis

Analysis of variance was employed to analyze experimental results using the generalized linear model procedures of SRISTAT2 for Windows software (Social Survey Research Information, Tokyo, Japan). Least-square means for each treatment were separated on the basis of least significant differences. Data are represented as mean ± SE for three cultures per treatment. Statistically significant differences at *P *<* *0.05 and *P *<* *0.01 are indicated by (*) and (**), respectively. Each experiment was repeated three times to verify the reproducibility of results.

## Results and Discussion

### Expression profiles of SDC members

The purpose of this study was to examine the nature of possible membrane receptor(s) for HGF and FGF2 which each impact Sema3A upregulation in early-differentiated satellite cells (myoblasts). Experiments were designed to test an alternate receptor hypothesis centered on the implication of a role for transmembrane-type proteoglycans (SDCs) through association of growth factors with heparan and chondroitin sulfate chains carried by the extracellular domain of core proteins.

**Figure 3:**
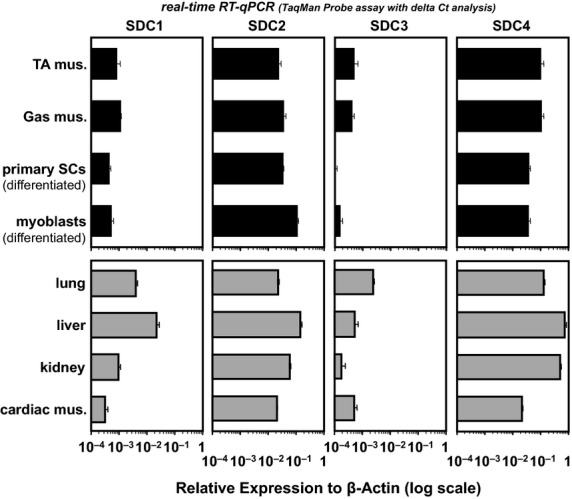
Expression profiles of SDC members in skeletal muscle, differentiated myoblasts, and referenced organs and tissues. Tibialis anterior (TA) and gastrocnemius muscles (Gas) of lower hind-limb, and cardiac muscle (not whole-heart tissue), lung, liver, and kidney were all collected from 10-week-old male C57BL/6J mice. Satellite cells (SCs) were isolated from muscle groups of upper hind-limb and back of the same mice and maintained in DMEM-10% HS until 72-h postplating – the early differentiation time point is as described previously (Tatsumi et al. 2009; Do et al. 2011). Mouse satellite cell-derived myoblasts (*myoblasts*) were also cultured for 72 h in DMEM-5% HS differentiation medium. Relative levels of mRNA expression of SDC members (SDC1–4) were determined by real-time RT-qPCR combined with the 2^–ΔΔC(t)^ analysis referenced to *β*-actin expression. Data bars depict the means ± SEs for three mice on a log scale.

Figure 3 displays the expression profiles of four SDC members (SDC1–4) in early-differentiation cultures of both satellite cell-derived myoblasts (at 72 h in differentiation medium; cell materials for the subsequent siRNA transfection experiments) and primary satellite cells (at 72 h postplating), as detected by RT-qPCR combined with delta-Ct analysis referenced to *β*-actin expression. This assay included tibialis anterior (TA) and gastrocnemius (Gas) muscles and other organs and tissues from adult mice (gray bars). These all served as reference controls, with their specific expression profiles highly comparable to those reported by Kim et al. (1994) in which relative abundance of each syndecan was determined by northern blotting and phosphorimager analysis of transcripts normalized to *β*-actin. Results by Kim et al. (1994) showed that SDC2 and 4 transcripts are highly abundant, whereas the levels of SDC1 and 3 expression were undetectable in adult C57BL mouse skeletal muscle. Similar SDC-mRNA profiles were seen in human cells (Lories et al. 1992) and mouse tissues and cells (Saunders et al. 1989; Brandan and Larrain 1998). By comparison, observations by Cornelison et al. (2001) using immunofluorescence staining, showed that SDC1, 3, and 4 proteins are all expressed in developing skeletal muscle tissue and that SDC3 and 4 proteins are highly restricted in adult mouse forelimb to cells retaining myogenic capacity (i.e., quiescent satellite cells). This qualitative study by Cornelison et al. (2001) also convincingly described SDC2 protein in connective tissue of mouse skeletal muscle, potentially accounting for the detection of SDC2 mRNA signals in tibialis anterior and gastrocnemius muscles and other organs examined here.

As shown in the upper panel of Figure 3, the transcripts for all SDC members were detected in both differentiated myoblast preparations (black bars of third and forth rows). Notably, it is evident that SDC2, 4 expression was highly abundant as in the case of whole muscle tissues, at a level two magnitudes higher than that of the SDC1, 3 subfamily. Brandan and Larrain (1998) have reported that the expression of SDC2 seems to be constitutive throughout differentiation of mouse myoblast cell line C2C12, in contrast to downregulation of SDC1 and 3. Similar expression profile of SDC2 was demonstrated in the differentiation cultures of C2C12 myoblasts by Gutierrez et al. (2006) as revealed by western blotting of the detergent-soluble fraction of the myoblasts and myotubes. Considered together with the observation by Cornelison et al. (2001), it is possible that SDC4 mRNA is expressed in both quiescent satellite cells and also in differentiated myoblasts and that SDC2 is expressed in differentiated myoblasts. Nonetheless, differentiation cultures of primary satellite cells (myoblasts soon after plating) and satellite cell-derived myoblasts have comparable expression profiles of SDC members. This provided an advantage that enabled substitution of satellite cell-derived myoblasts for primary satellite cell cultures in the subsequent SDC knockdown experiment, as a validated and fast-track approach to evaluate the possible role of SDCs as the membrane receptor responsible for Sema3A upregulation exclusively at the early-differentiation phase.

### Possible implication of SDC2 and 4 in Sema3A upregulation

**Figure 4:**
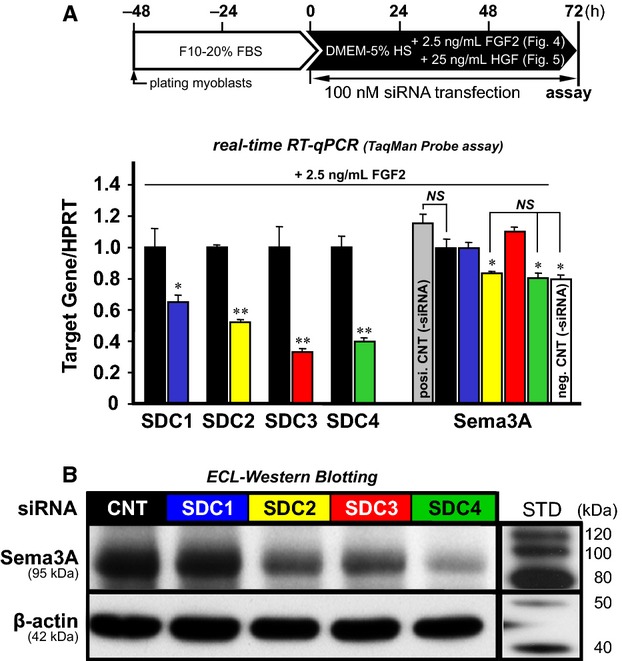
SDC2, 4 may mediate FGF2-induced Sema3A upregulation in myoblasts. Myoblast cultures were transfected with each siRNA at 100 nmol/L, as follows: control siRNA (black bars) and SDC1–4 siRNA (colored bars; Stealth_384, 715, 789, and 251, respectively) in DMEM-5% HS with 2.5 ng/mL FGF2 (see an upper scheme shown in panel A for the culture design), followed by evaluation for expression levels of SDC1–4 and Sema3A at 72 h by RT-qPCR (panel A) and western blotting (panel B; a representative cell lysate sample from three cultures per treatment group normalized to *β*-actin expression levels). Note that there were no significant differences (NS) in Sema3A expression levels between the negative control (open bar, without siRNA and FGF2 addition) and the cultures transfected with SDC2, 4 siRNAs, as well as between the positive control (gray bar, treated with 2.5 ng/mL FGF2 without control siRNA) and the experimental control cultures (black bars and CNT, treated with both 100 nmol/L control siRNA and 2.5 ng/mL FGF2). Data bars depict the means ± SEs for three cultures per treatment and significant differences from the experimental control culture mean at *P* < 0.05 and *P *<* *0.01 are indicated by (*) and (**), respectively. STD, molecular weight standards.

**Figure 5:**
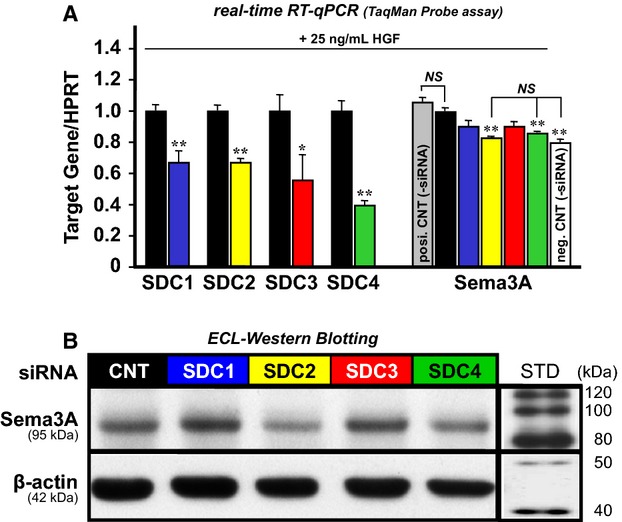
SDC2, 4 may mediate HGF-induced Sema3A upregulation in myoblasts. Myoblast cultures were transfected with each siRNA at 100 nmol/L, as follows: control siRNA (black bars) and SDC1–4 siRNA (colored bars; Stealth_384, 715, 789, and 251, respectively) in DMEM-5% HS with 25 ng/mL HGF (the same culture design as Fig. 4), followed by evaluation for expression levels of SDC1–4 and Sema3A at 72 h by real-time RT-qPCR (panel A) and western blotting (panel B; a representative sample from three cultures per each treatment group normalized to *β*-actin expression levels). Note that there were no significant differences (NS) in the level of Sema3A expression between the negative control (open bar, without siRNA and HGF addition) and the cultures transfected with SDC2, 4 siRNAs, or between the positive control (gray bar, received 25 ng/mL HGF without control siRNA) and the experimental control cultures (black bars and CNT, received both 100 nmol/L control siRNA and 25 ng/mL HGF). Data bars depict the means ± SEs for three cultures per treatment and significant differences from the experimental control culture mean at *P *<* *0.05 and *P *<* *0.01 are indicated by (*) and (**), respectively. STD, molecular weight standards.

In order to examine if SDCs implicate in Sema3A expression signaling, satellite cell-derived myoblasts were transfected with SDC-targeting Stealth siRNAs in differentiation medium (DMEM-5% HS) additionally containing each Sema3A upregulator, FGF2 (2.5 ng/mL) or HGF (25 ng/mL), and cultures were assayed for expression levels of Sema3A by RT-qPCR at 72 h (Figs. 4 and 5). Knockdown efficiencies here (about 30–70%) were lower than the siRNA silencing potential (about 80%; see Fig. 2 again), likely due to the longer period of differentiation in culture (72 h vs. 24 h) that resulted in more cell growth and hence lower transfection performance. It is evident that SDC2 siRNA (stealth_715; yellow bars) and SDC4 siRNA (stealth_251; green bars) significantly decreased the expression of Sema3A message down to a level comparable to the negative control culture that did not receive either siRNA or one of the growth factors (open bars). By comparison, transfection with SDC1, 3 siRNA (blue and red bars) did not significantly reduce Sema3A expression, as shown in the right columns of Figs. 4A and 5A. These responses were more evident at the Sema3A-protein level, as revealed by western blotting of whole cell lysates standardized with internal *β*-actin (Figs. 4B and 5B). Positive control cultures treated with 2.5 ng/mL FGF2 or 25 ng/mL HGF (gray bars in Figs. 4A and 5A) showed a level of Sema3A expression similar to the control siRNA cultures with each growth factor (black bars in the right columns; not significantly lower) and significantly higher than the mean for negative control cultures (open bars, without siRNA and the growth factors). Hence, these growth factor-treated cultures served as an important control group to confirm the expected activity of each growth factor under the present experimental design. The response to growth factors seen as a 20–40% increase in Sema3A mRNA expression was smaller (but statistically significant at *P *<* *0.05) than that in typical primary cultures without siRNA lipofection (see Refs. Do et al., (2011), Tatsumi et al., (2009)), possibly due to the difference in cell materials used and negative side-effects of active surfactants formulated in the lipofection reagents, on functional activities of membrane receptors. Nonetheless, our concurrent results demonstrated that knockdown of transmembrane proteoglycans SDC2 and 4 abolished FGF2/HGF-induced Sema3A upregulation, reducing it to the baseline level, therefore indicating that SDC2, 4, but not SDC1, 3, may mediate the FGF2/HGF-signaling pathway that induces Sema3A expression exclusively at the early differentiation phase of myoblasts. The exuberant expression of the SDC2, 4 subfamily relative to SDC1, 3 in early-differentiated myoblasts provides support for this conclusion.

### Hypothetical signaling mechanisms

**Figure 6:**
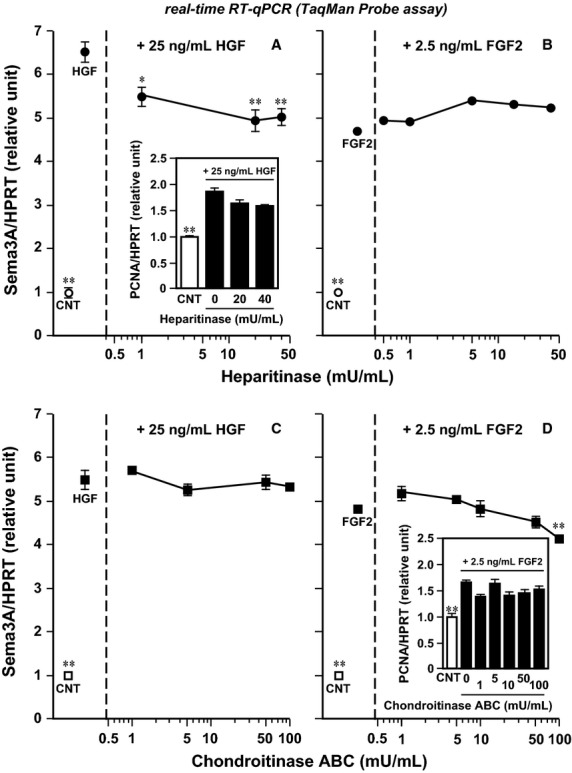
Positive effects of glycosaminoglycan chain hydrolysis on HGF/FGF2-induced Sema3A upregulation. Primary cultures of rat satellite cells were maintained in DMEM-10% HS for 48 h and then pretreated with one of the GAG-chain hydrolyzing enzymes, heparitinase (0.5–40 mU/mL) from *Flavobacterium heparinum* (100703; Seikagaku, Tokyo, Japan) (A, B) or chondroitinase ABC (1–100 mU/mL) from *Proteus vulgaris* (100332; Seikagaku) (C, D) in serum-free DMEM, for 90 min before a 24-h period of supplementation with 25 ng/mL HGF (A, C) and 2.5 ng/mL FGF2 (B, D). Cell lysates were collected and analyzed for expression of Sema3A (closed symbols) and proliferating cell nuclear antigen (PCNA; panels A and D, insets) by real-time RT-qPCR standardized with HPRT. Open symbols and bars labeled “CNT” (far left) represent negative control cultures without any treatment. Closed symbols labeled “HGF” and “FGF2” represent positive control cultures without enzyme treatment and with 25 ng/mL HGF and 2.5 ng/mL FGF2, respectively. Data points and bars depict the means ± SEs for three cultures per treatment and significant differences from each positive control culture mean at *P *<* *0.05 and *P *<* *0.01 are indicated by (*) and (**), respectively. Note that the heparitinase/chondroitinase ABC treatments do not significantly disturb the cell viability (*P *>* *0.05) as monitored by the mRNA expression level of PCNA, in the enzyme–dose ranges examined (panels A and D, insets).

There is still a large gap in our understanding of how SDC2 and 4 generate the Sema3A expression signal in response to HGF and FGF2 binding, although SDCs have an affinity for both growth factor ligands on their heparan sulfate and chondroitin sulfate chains that are carried by the extracellular domain of core proteins (Carey 1997; Rapraeger 2000; Asada et al. 2009; Xian et al. 2010) and the intracellular domain possesses well-conserved tyrosine residues potentially responsible for the signal transduction (Carey 1997; Multhaupt et al. 2009; Xian et al. 2010; Choi et al. 2011). The present study does not show significant evidence on this important issue. However, a supplementary contribution to address this question may be found in our preliminary results (Fig. 6), which show that pretreatment with heparitinase diminished HGF-induced Sema3A upregulation (but not the response to FGF2) in cultures of differentiating satellite cells (panels A and B), while chondroitinase ABC pretreatment did diminish the response to FGF2 (but not to HGF; see panels C and D). These findings led to the idea that HGF and FGF2 may bind to the heparan sulfate and chondroitin sulfate chains, respectively, of SDC2 and 4 to initiate the Sema3A expression signaling. This model is supported by comparative and biochemical studies that demonstrated that SDC4 carries both heparan sulfate and chondroitin sulfate chains in normal human saphenous vein endothelial cells and a clonal L cell line (Shworak et al. 1994; Carey 1997) and normal murine mammary gland epithelial cells (Deepa et al. 2004; Tkachenko et al. 2005), similar to SDC1 (Ueno et al. 2001). SDC2 and 3 are not reported to carry chondroitin sulfate chains in a variety of tissues and cells including neonatal rat brain, heart, and Schwann cells (Carey et al. 1992) and developing chicken brain and neural tube (Gould et al. 1995; Kosher 1998).

**Figure 7:**
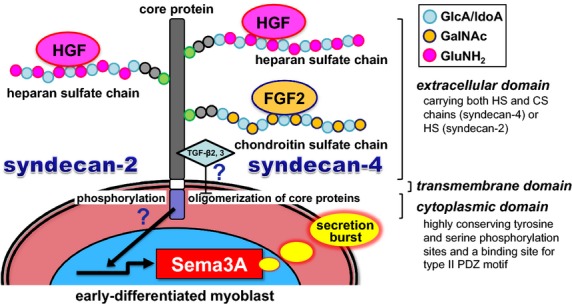
Hypothetical signaling mechanisms for HGF/FGF2-induced Sema3A upregulation in early-differentiated myoblasts. HGF and FGF2 could associate with the heparan sulfate chain of SDC2, 4 and the chondroitin sulfate chain of SDC4, respectively, to form HGF-SDC2, 4 and FGF2-SDC4 complexes (functionally equivalent), which could activate a signaling cascade of events that includes oligomerization of core proteins as a crucial step to activate phosphorylation of the intracellular tyrosine residues. TGF-*β*2, 3 binding to the ectodomain of SDC2, 4 could inhibit (and dissolve) the oligomerization of core proteins and hence turns off the Sema3A expression signaling. This model extends our understanding of a new concept of regenerative motoneuritogenesis that is centered on the functional coupling of M2 macrophages (a paracrine source of the ligands at the early differentiation phase) with myoblasts (via activation of the SDC2, 4 receptor and induction of Sema3A secretion) and a motoneuron terminal (Sema3A target). This coupling may coordinate a delay in the attachment of motor neuron terminals onto damaged and regenerating new fibers early in myogenic repair, and thus synchronize the recovery of nerve–muscle integrity.

Considering together our previous important observations that the HGF/FGF2 responses share a similar dose-dependent character without any additive or synergistic effects of the two growth factors together (Tatsumi et al. 2009; Do et al. 2011), HGF and FGF2 could associate with the heparan sulfate chain of SDC2, 4 and the chondroitin sulfate chain of SDC4, respectively, to form HGF-SDC2, 4 and FGF2-SDC4 complexes that may be functionally equivalent (see a hypothetical model depicted in Fig. 7). Those complexes could activate a signaling cascade of events that includes noncovalent, homologous oligomerization of core proteins (promoted by a GG motif conserved in the SDC transmembrane domain) as a crucial step to activate kinase activity and phosphorylation of the intracellular tyrosine residues upon ligand binding (Choi et al. 2005, 2011; Xian et al. 2010). By contrast, epidermal growth factor (EGF) has only a low potential to induce Sema3A expression in culture (about 30% relative to HGF at the same concentration of 25 ng/mL), and other growth factors including insulin-like growth factor-1 (IGF-1), platelet-derived growth factor-BB (PDGF-BB), and transferrin, found in crushed muscle extract and involved in postnatal myogenesis (Bischoff 1986; Chen and Quinn 1992; Chen et al. 1994; Tatsumi et al. 1998; Anderson and Pilipowicz 2002), have no effect on Sema3A expression (Tatsumi et al. 2009). These reports support the above “limited-alternative mode” of interaction between HGF/FGF2 ligand proteins and heparan/chondroitin sulfate chains of SDC2, 4.

Consistent with these considerations, it is worth noting that both TGF-*β*2, 3 prevent HGF/FGF2-induced Sema3A upregulation in cultures (Do et al. 2011) and that TGF-*β* directly binds to the ectodomain of SDC2 core protein (Chen et al. 2004). It is possible that TGF-*β*2, 3 binding inhibits (and dissolves) the oligomerization of core proteins, and hence turns off the Sema3A-expression signaling that is triggered by association of HGF and FGF2 to the corresponding GAG chains (heparan and chondroitin sulfate chains, respectively) of SDC2, 4. This would result in the downregulation of Sema3A expression to a baseline level. This supplementary model may fall into place as a physiological explanation for our observation that the level of active TGF-*β*3 increases with a delay (about 4 days) after the peak prevalence of HGF and FGF2 in extracellular wound fluids after crush injury of gastrocnemius muscle (Do et al. 2012). As such, the model adds to our understanding of the time-coordinated levels of HGF and FGF2 (upregulators) and TGF-*β*3 (downregulator) that drive temporal regulation of Sema3A expression during regenerative intramuscular motor neurite formation (motoneuritogenesis), although the detailed inhibitory mechanisms and evidence of the phosphorylation of SDCs after FGF2 and/or HGF binding still await further study.

In conclusion, this study showed that the transmembrane proteoglycans, syndecan-2, 4 (SDC2, 4), could function as membrane receptors for HGF and FGF2 ligands in signaling expression of the neural chemorepellent Sema3A, in early-differentiated myoblasts. Although further studies are required to elucidate the detailed molecular mechanisms including SDC2, 4 phosphorylation upon ligand binding and the downstream signaling pathways, heparan and chondroitin sulfate chains are now expected to be primary elements of the HGF and FGF2 binding sites in SDC2, 4 ectodomains. The present findings, therefore, extend our understanding of a new concept of regenerative motoneuritogenesis that is centered on the functional coupling of M2 macrophages (a paracrine source of the ligand, HGF at the early differentiation phase; see Shono et al. 2013; Sakaguchi et al. 2014; Sawano et al. 2014) with myoblasts (via activation of the SDC2, 4 receptor and induction of Sema3A secretion) and a motoneuron terminal (Sema3A target). This coupling of HGF/FGF2-SDC2, 4 and Sema3A may coordinate a delay in the attachment of motor neuron terminals onto damaged and regenerating new fibers early in myogenic repair, and thus synchronize the recovery of nerve–muscle integrity. Since both Sema3A and SDCs are expressed in a variety of organs and tissues (Kim et al. 1994; Roth et al. 2009; Xian et al. 2010), the HGF/FGF2-SDC2, 4-Sema3A pathway could possibly function in the regulation of angiogenesis, osteogenesis (acting on osteoblasts and osteoclasts), organogenesis, immune responses, and tumor progression, all of which have been studied for the essential contributions of Sema3A expression (Miao et al. 1999; Serini et al. 2003; Lepelletier et al. 2006, 2007; Hayashi et al. 2012; Fukuda et al. 2013). Considering these crucial functions of Sema3A and SDC2, 4, the present study may contribute to finding new medications that specifically promote repair from muscle injury, including traumatic lesions that affect motor axons, and also from neuromuscular disorders such as amyotrophic lateral sclerosis (ALS) and murine motor end-plate disease. New information would also be conducive to finding a novel strategy to combat the progressive loss of motor innervation that is often seen in age-related atrophy, sarcopenia.
